# Online Communication and Body Language

**DOI:** 10.3389/fnbeh.2021.709365

**Published:** 2021-09-07

**Authors:** Paolo Paradisi, Marina Raglianti, Laura Sebastiani

**Affiliations:** ^1^Istituto di Scienza e Tecnologie Dell'Informazione “A. Faedo” (ISTI-CNR), Pisa, Italy; ^2^BCAM-Basque Center for Applied Mathematics, Bilbao, Spain; ^3^Departamento de Educación de la Facultad de Educación y Humanidades, Universidad de Tarapacá, Arica, Chile; ^4^Department of Translational Research and New Technologies in Medicine and Surgery, University of Pisa, Pisa, Italy

**Keywords:** COVID-19, interpersonal online communication, perceived interpersonal distance, dance movement therapy, body movements, mirroring, social interactivity, cooperation

## Introduction

The COVID-19 emergency brought out the role of online digital technologies. The increase in online social interactivity was accelerated by social distancing, which has been recognized to have adverse effects due to physical and emotional isolation (Canet-Juric et al., 2020).

Body language is central to social interactions, and its role is clearly diminished when going online, but the relevance of this change is still not clear. This transition toward online could affect the wellness of the people, especially the population with specific fragilities, e.g., young people and seniors (Beam and Kim, 2020; Canet-Juric et al., 2020; Fernández Cruz et al., 2020).

We here briefly present our viewpoint on some issues concerning changes in body interactions in online interpersonal communication. Our aim is to encourage constructive discussion and raise awareness about these very topical issues.

## Online Communication Platforms: Toward New Communication Strategies?

Progress in digital technologies is having a profound impact on interpersonal communication. The natural face-to-face modality is nowadays often replaced by interactions through video-mediated online communication platforms (VMOCPs) (mediatized communication). Furthermore, the duration of restrictions related to the COVID-19 emergency determined, in a very short time, an acceleration in the diffusion and an intensification in the use of VMOCPs.

In fact, VMOCPs are now ubiquitously used for meetings and courses in different contexts, such as work environments, education, and, in general, for whatever activity involving social interaction, thus, determining rapid changes in the everyday lives of the people (Chan et al., 2020; Dorn, 2021).

Interestingly, also the seniors, which were only marginal users of these technologies, were forced to use them as their only chance for social contacts (López et al., 2020; Pelicioni and Lord, 2020).

The new form of communication has brought great improvement in communication possibilities, by overcoming the limitations of time and space. However, VMOCPs have also modified the communication rules, e.g., those related to proxemics (Hall, 1966). Proxemics assumes a direct proportionality between the geometrical peripersonal/extrapersonal space and different types of interpersonal acquaintanceship: intimate, personal, social, and public. When people communicate through VMOCPs, the geometrical distance separating the screen image and the real interlocutor is a few tens of centimeters, which is smaller than the distance between persons involved in a social/public face-to-face conversation. Then, when passing online, does a short “perceived” distance imply a personal/intimate level? In our opinion, the answer to this question is not obvious as going online likely brings non-trivial changes in the proxemics rules.

For example, such a physical closeness would presuppose an intimacy between persons and a mutual disposition to the potential use of the tactile channel (handshake, hug, and tap on the shoulder). However, despite this virtual closeness, we cannot touch (Drag, 2020).

Another important change concerns the communicative role of eye gaze. In fact, in agreement with the “fractured ecologies” concept (Heath and Luff, 1992; Luff et al., 2003), the eye contact and the meaning it conveys (Drag, 2020), when mediated by the camera, is unrecoverable by the interacting participants.

In summary, the above discussed issues suggest that changes from live to online communication are complex, as the direct proportionality between (apparent) geometrical distance and interpersonal acquaintanceship is deeply changed both quantitatively and qualitatively.

## Body Motion and Video-Mediated Online Communication Platforms: A Focus on Online Education and Therapeutic Intervention

Body movements and language are crucial in both emotion-based non-verbal communication and cognitive-based social interactions. It is foreseeable that extensive use of online technologies could have important effects on cognitive processes, not only those involving learning/educational/training activities but also those related to emotion-driven relationships in social living. To date, however, the role of body movements in inter-subject online interaction has been scarcely investigated (Zuo et al., 2021).

Let us discuss two different examples: online education and dance movement therapy (DMT).

Serious games are interactive virtual simulations whose goal is to train while entertaining. They were proposed as useful tools to improve learning performances (Hanus and Fox, 2015) and, with the COVID-19 emergency, have been thought as a possible mainstream solution to mitigate the problem of social distancing in online teaching (Koivisto and Hamari, 2019).

This approach, although certainly useful, needs to be reviewed considering the concept of “embodied education,” which refers to the embodied cognition (EC) theory (Kiverstein and Miller, 2015; Shapiro, 2019). According to it, cognition is formed by the processing of informative stimuli from and through the body. Some authors found a positive correlation between learning performances and movement synchronization in the teacher–learner interaction (Sacheli et al., 2018; Shapiro and Stolz, 2019; Madsen et al., 2020; Pan et al., 2021). Furthermore, mobility strategies of the teachers in the classroom (classroom proxemics) at various stages of a lesson and according to the task of the students have been found to have positive effects on engagement (Chin et al., 2017), motivation (Fernandes et al., 2011), and disruptive behavior of the students (Gunter et al., 1995).

DMT is a complementary therapy where body movements are employed to promote personal and social wellbeing. This is reached by eliciting the harmonization of mental, somatic, and relational manifestations of the individual through the creative use of movement improvization and dance (ADTA—American Dance Therapy Association, 2014). DMT emphasizes the communicational aspect of dance (Karkou and Sanderson, 2006). In fact, the DMT setting presupposes a social component triggered by interpersonal interactions mediated by body language and, in particular, movements. Indeed, the social component through body interaction has a crucial role in DMT functioning: the game of distances, perspectives, and reciprocity creates the communicative context in which movement takes place. The circle, for example, is a DMT basic figure (Karampoula and Panhofer, 2018) in which all group members can see everyone else, thus, having direct access to verbal and nonverbal cues of the participants. The basic elements of a circle include mirroring, echoing of emotional states, containment and holding, and physical contact through the holding of hands. Mirroring consists of matching/echoing the movements of the person (Tortora, 2006) and, in a circle, is multiple (multimirror). This technique has been shown to be effective in strengthening the self-confidence and physical resilience of the group members but also social integration and affiliation by promoting empathy. The “motor theory of empathy,” in fact, proposes that the human mirror system may participate to the understanding of the intentions and feelings of others (Rizzolatti and Fabbri-Destro, 2008) and that empathy may stem from the link between perception and action (Iacoboni, 2009; Zardi et al., 2021). In brief, understanding of action may promote empathizing with others (Carr et al., 2003). Despite this theory being criticized (Hickok, 2014), phenomena based on motor resonance (i.e., a direct link between the perception of an action and its execution), namely, mimicry, synchrony, and automatic imitation, are considered involved in higher social cognition, including empathy, and in promoting positive social effects both in the adult and during development (Rauchbauer and Grosbras, 2020).

## What Is Missing Online?

In DMT, the dyadic relationship between the conductor/participant is at least partially preserved (APID—Associazione professionale italiana danzamovimentoterapia, 2021). The conductor can stimulate the participant with the voice, music, and gestures also through the screen. The participant, by observing the conductor on the screen, can be accompanied in the experimentation of his own movement aimed at creating an internal/external dialog. Indeed, evidence from trials of online meditation (Cavalera et al., 2019; Yang et al., 2019) supports the idea that online, it is possible to work on the “vertical dimension of energy” that is on the individual depth of feelings. In contrast, the transversal level, which includes all the non-verbal interactions between participants, is greatly impaired. Even with groups of three to four persons, most of the interpersonal and transpersonal components of movement cannot be reproduced.

The example of DMT shows that online sensory interaction is very different from a live one. The visual and auditory sensory channels are essentially the only online communication modalities. Actually, images on the screen are two-dimensional (2D), thus, reducing tridimensional visual perception to a *quasi* 2D one^1^. Other modalities such as the tactile and olfactory ones cannot be directly employed. Human touch has been suggested to play a large role in establishing a sense of “proximity” between persons and to facilitate affiliative behavior and social bonding (Morrison et al., 2010). Previous studies have demonstrated a close association between pleasant social touch and the release of oxytocin, which is a crucial modulator of social behavior and emotions across species (Olff et al., 2013; Walker et al., 2017; Kendrick et al., 2018; Tang et al., 2020).

The sense of smell is also involved in the non-verbal social communication of humans; in fact, through smell, humans can involuntarily convey personal information (de Groot et al., 2017; Parma et al., 2017; Pause, 2017; Roberts et al., 2020).

Some authors claim that chemosignal communication critically contributes to the formation and maintenance of social groups and has a role in the evolution of the social brain (Dunbar and Shultz, 2007; Parma et al., 2017).

A new multidisciplinary discipline involving psychology and chemistry (sociochemistry) is studying the chemical basis of olfactory communication emphasizing the role of psychological states and traits in modulating body odorant composition (de Groot et al., 2020). Interestingly, data from patients with no or reduced olfactory capabilities show that the loss of smell severely affects the richness of social relationships over the lifespan from the early developmental stages to the old age (Boesveldt et al., 2017).

## Final Discussion

We can argue that in online social interactions:

(i) smell and touch are absent;
(ii) visual is limited to a *quasi* 2D perception;
(iii) auditory is almost unchanged;
(iv) changes in the relationship between perceived geometrical distances and acquaintanceship
  are expected, but still unclear;
(v) there are no direct bodily interactions.

According to the “fractured ecologies” concept, when online, interacting persons cannot recover most of the relevant features of the environment and bodily behavior of others (gesture, eye contact) and behave accordingly (Luff et al., 2003).

These changes may undermine the emotional and empathetic aspects of interpersonal communication. However, cooperation is still possible through the auditory and the reduced *quasi* 2D visual perception.

A better understanding of these aspects could need a partial revision of classical communication theories (McLuhan, 1964; Hall, 1966; Bolter and Grusin, 1999; Jensen, 1999) in order to consider the new modalities of communication introduced by online interactions. Thus, the consequences of passing online probably include *remediation* mechanisms related to the use of the new digital technologies (Bolter and Grusin, 1999). These mechanisms are compatible with the *self-organization* paradigm of complexity: cooperative social dynamics between different individuals/groups trigger the emergence of a new dynamical equilibrium in a relatively short time due to an environmental change (in this case, the new digital technologies) (Zeleny, 1977; Santos et al., 2006; Paradisi et al., 2015; Paradisi and Allegrini, 2017; Mahmoodi et al., 2018). The equilibrium is constrained to the optimization of social interactivity mediated by the new technology, the optimum possibly being a condition as nearest as possible to live interactivity. Thus, the question that should be answered should not only be “what is lost?” but also “what is new?,” possibly involving the development of *virtual proxemics*.

An open question that deserves further investigations is the quantification of perceived virtual distances in online interactions. For example, the distance between the screen and the individual could be used, but the effect of the image size on the screen should also be clarified.

**Figure 1:**
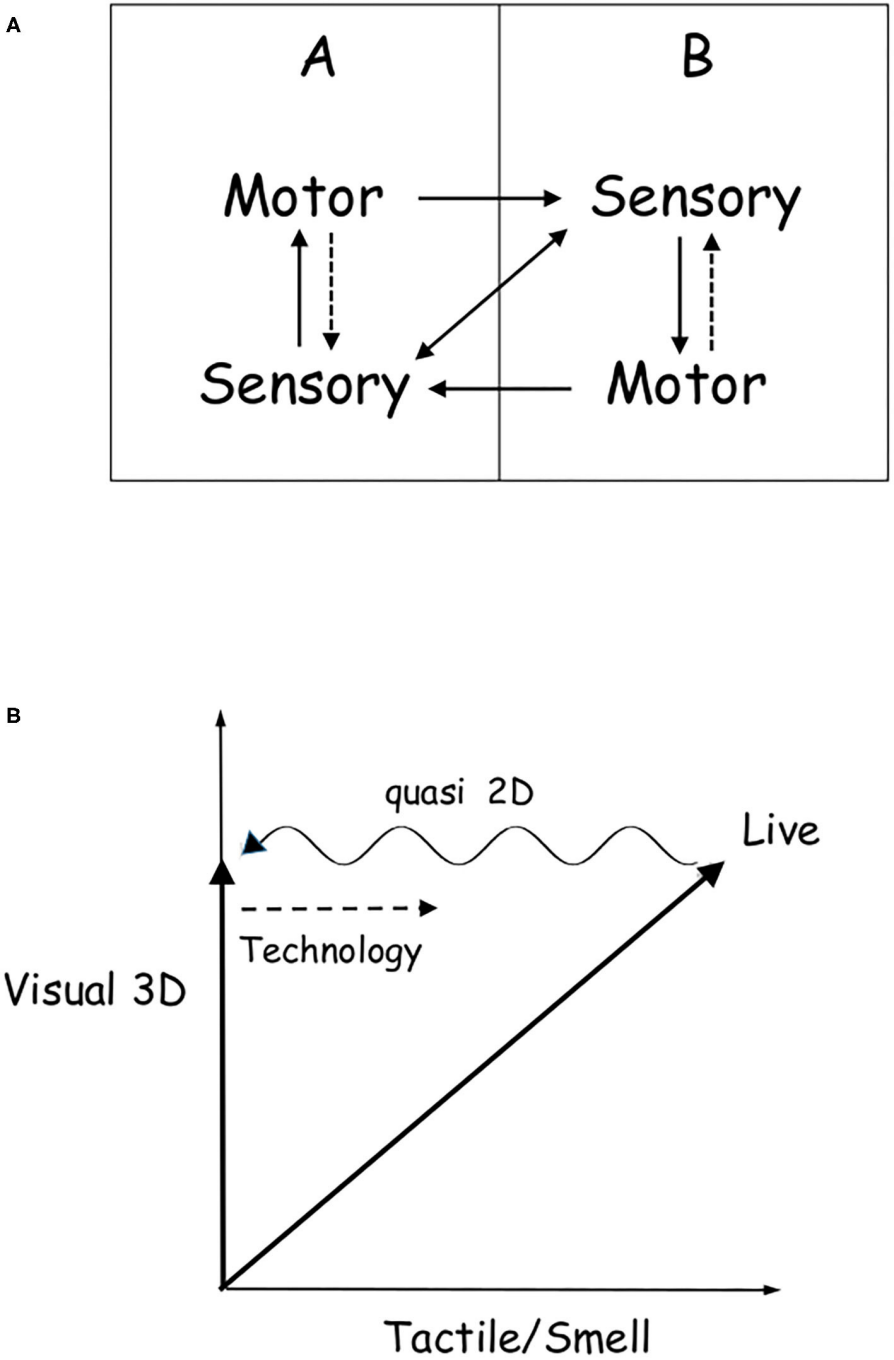
**(A)** Simple scheme of a live interaction between individuals A and B. The two horizontal arrows indicate the mutual feedback between A and B, while the vertical ones denote the individual internal information exchanges between the sensory and motor systems. The vertical motor-to-sensory dashed arrows indicate the increased strength of sensory-to-motor (continuous arrows) when an individual interacts, thus, triggering the clockwise feedback loop, which represents the emergence of self-organizing behavior of A–B as a whole. The double diagonal arrow represents the direct interaction through the sensory system, e.g., eye contact or touch. **(B)** Qualitative sketch of the passage from live to online interactions. Live interactions involve both visual and tactile/smell senses, while online determines a reduced perception, represented in the figure as a shift toward the *quasi* 2D vision. The wave in the left arrow denotes that the projection on the vision axis is neither two- nor three-dimensional, but in between. The right dashed arrow represents technologies needed to (partially) fill the online–live interactivity gap. Being almost unchanged, auditory is not reported.

Figure 1 reports a graphical summary of our discussion. Panel (A) is a scheme of self-organizing live interaction between two individuals. Panel (B) sketches the passage from live to online interactions.

To address the challenges of online communications and to mitigate the effect of emotional isolation, an interdisciplinary research is needed that would have to (i) monitor the social change, (ii) develop new communication models, and (iii) develop strategies and technologies to partially fill the online-live interactivity gap.

## Author Contributions

PP and LS wrote the paper and carried out the bibliographic search with many insights from MR regarding DMT and body language in social communication. All authors equally discussed and developed the idea of the paper.

## Conflict of Interest

The authors declare that the research was conducted in the absence of any commercial or financial relationships that could be construed as a potential conflict of interest.

## Publisher's Note

All claims expressed in this article are solely those of the authors and do not necessarily represent those of their affiliated organizations, or those of the publisher, the editors and the reviewers. Any product that may be evaluated in this article, or claim that may be made by its manufacturer, is not guaranteed or endorsed by the publisher.

## Footnotes

^1^Here “quasi” is related to a partial mental reconstruction of vision depth that the brain can operate on the basis of past live experiences, by elaborating what is actually a two-dimensional image on the screen.
